# The Significance of Genetic Polymorphisms within and between Founder Populations of *Ceratitis capitata* (Wied.) from Argentina

**DOI:** 10.1371/journal.pone.0004665

**Published:** 2009-03-02

**Authors:** Alicia Basso, Laura Martinez, Fanny Manso

**Affiliations:** 1 Cátedra de Genética, Facultad de Agronomía, Universidad de Buenos Aires, Buenos Aires, Argentina; 2 Laboratorio de Insectos, Instituto de Genética “Ewald A. Favret”, CNIA-Instituto Nacional de Tecnología Agropecuaria, Castelar, Argentina; University of Exeter, United Kingdom

## Abstract

**Background:**

The Mediterranean fruit fly *Ceratitis Capitata* (DIPTERA: Tephritidae) is a major agricultural pest in Argentina. One main cause for the success of non-contaminant control programs based on genetic strategies is compatibility between natural and laboratory germplasms.

A comprehensive characterization of the fruit fly based on genetic studies and compatibility analysis was undertaken on two founder populations from the provinces of Buenos Aires and Mendoza, used in pioneering sterile male technique control programmes in our country. The locations are 1,000 km apart from each other.

**Methodology/Principal Findings:**

We compared the genetic composition of both populations based on cytological, physiological and morphological characterization. Compatibility studies were performed in order to determine the presence of isolation barriers. Results indicate that the Buenos Aires germplasm described previously is partially different from that of the Mendoza population. Both laboratory colonies are a reservoir of mutational and cytological polymorphisms. Some sexual chromosome variants such as the XL and the YL resulting from attachment of a B-chromosome to the X-chromosome or Y-chromosome behave as a lethal sex-linked factor. Our results also show incompatibility between both germplasms and pre-zygotic isolation barriers between them. Our evidence is consistent with the fact that polymorphisms are responsible for the lack of compatibility.

**Conclusions:**

The genetic control mechanism should be directly produced in the germplasm of the target population in order to favour mating conditions. This is an additional requirement for the biological as well as economic success of control programs based on genetic strategies such as the sterile insect technique. The analysis of representative samples also revealed natural auto-control mechanisms which could be used in modifying pest population dynamics.

## Introduction

The Mediterranean fruit fly (medfly), *Ceratitis capitata* (Wiedemann), is the most economically important agricultural pest insect in the world. It belongs to the Tephritidae family, the “true fruit flies”, which is the target of large-scale eradication and suppression programs based on genetic strategies like the sterile insect technique (SIT)[1], [2]. A transgenic strain was created in the medfly [3] that could be used as a redundant back-up for or replacement of sterilization by irradiation, either on its own or in combination with the genetic sexing strains [3] already constructed by classical genetics [4].

At present a particular transgenic strain of the medfly [5] is being supplied to many operational SIT programs worldwide to control natural populations [6].

Reared medflies must display morphological, physiological and behavioural features that are as close as possible to those of their wild counterparts. Any departure from the “wild” characteristics could cause the failure of an SIT program [7].

Considerable genetic variation in natural populations of the medfly from different geographic regions has been previously reported by different researchers [8]–[19]. The presence of actively transposing elements in the medfly genome is revealed by hybrid dysgenesis phenomena, insertion site polymorphisms and other genetic instabilities [20]–[23]. Part of the problem is to understand the significance of genetic variability within and between insect populations.

A comprehensive characterization of the fruit fly based on genetic studies and compatibility analysis was undertaken on two founder populations from the provinces of Buenos Aires and Mendoza. These materials were used in pioneering sterile male technique control programs in our country.

The success of non-contaminant control methods based on genetic strategies depends on compatibility between natural and laboratory germplasms.

The utility of insect colonies depends on the laboratory conditions in which they are established and the precision with which they are managed. In fruit fly colonies, large and genetically variable founder populations are collected and carefully maintained in laboratory environments that provide -according to the investigator's criteria- optimal quantities and qualities of diet and space so as to promote the highest possible levels of survival for all developmental stages. However, some genotypes are lost, and this is not always a consequence of rearing.

At least three unmanageable events contribute to genetic drift in laboratory colonies of *C. capitata* (Wied.).

1. Sampling itself, especially if sample size is small, could favour genetic drift. The medfly *C. capitata* is a “polyphage” and “multivoltine” species, so, generally, founder karyotypes do not really represent the whole genetic pool of the natural population. The collection of samples is mainly performed in the most economically important host-fruits during the same period every year. Limited knowledge of the biology and oviposition strategies of the fruit fly in nature, and within economically unimportant host-fruits, is further obstacle to improving the collection of samples. For this reason, we are probably losing a great deal of the natural genetic variability, since there may be different genetic associations between this fruit fly and other host fruits that are economically unimportant.
2. Differential adaptation of the founder genotypes: laboratory conditions fit some genotypes but not others.
3. Evolutionary processes caused either by accidental changes in laboratory conditions or by mutation events, which are beyond control. These changes are a consequence of rearing and can be studied because: a) there is a large amount of individuals per generation; b) the life cycle of fruit flies is shortened and the number of generations per year increases.

The importance of analyzing genetic variability was demonstrated in the screw-worm *Cochlyomia hominivorax* (Coquerel). A program to eradicate this pest in the United States, based on the release of sterile blowflies, failed because a state of reproductive incompatibility developed between wild-type and laboratory-reared individuals. Later on, a chromosomal polymorphism affecting the genital morphology of wild type females was associated with isolation barriers [24]. Invasions of medfly in a modern global trade network tend to be due to multiple introductions. This fact allows a maintenance or enhancement of genetic variability in the adventive populations, which in turn increases their potential invasiveness [19].

Previous work performed in our laboratory demonstrated the existence of different chromosomal polymorphisms within geographic populations from the provinces of Buenos Aires, Tucumán, Mendoza and Río Negro [8]–[12]. Genetic polymorphisms within a Buenos Aires colony named *ARG 17* have been studied through the years. Next, a picture of this variability is summarised. Variation in the number of internal orbital bristles or spatulated hairs was observed in males. It was determined that in females, a gene is responsible for the increase in rostrum orbital hairs. It was demonstrated that these genes have a pleiotropic effect and variable expression [8]. Both the electrophoretic pattern and the inheritance of the first allozyme locus described in the species – the *Est-1* gene, a pupal esterase with two codominant alleles – was reported by some authors [25].

Polymorphisms, named Y_A_ and Y_B_ and affecting the long arm of the Y-chromosome, were reported, but they alter neither the sexual determining factor nor the fertility of carrier individuals [26]–[28]. In 1995, the frequencies of Y_B_ and Y_A_ chromosomes were 0.6 and 0.4 [12] respectively.

A polymorphism affecting the length of the X-chromosome is present within a stock originated in the same ecological niche as *ARG 17* [11]. The variant -named X_L_- is derived from the attachment of a B-chromosome to the X_S_ chromosome, and its inheritance was also reported by these authors. Homozygous female X_L_X_L_ were never found [11].

One of our studies showed isolation barriers between individuals from this laboratory (origin: Province of Buenos Aires) and those from a laboratory colony in the Province of Mendoza [10]. The study revealed: 1) incompatibility between both populations evidenced by a drop in the percentage of fertile mates; 2) the dominant expression in the F_1_ offspring, of an allele previously described as a recesive one; 3) individuals from the Mendoza population showed a high frequency of chromosomal polymorphism; 4) 16% of the chromosomes tested by backcrossing showed distorted segregation.

Colonies from Buenos Aires and Mendoza have been periodically analyzed and used in pioneering sterile male technique programs to control *C. Capitata* in our country. One of them is from North Central Buenos Aires province – in the plains region, with a temperate, rainy climate. The other colony comes from the province of Mendoza, which is in an artificial oasis in a pre-Andean desert area. The locations are 1,000 km apart from each other. The purpose of our present study is to analyze the structure of both Argentinian *C. capitata* founder populations from different geographic origins. This is to determine whether they are compatible, and establish if their cytological and/or morphological complexities can account for the lack of compatibility. The analysis is based on the description of physiological behaviors and chromosomal polymorphisms as well as their possible associations. In keeping with this design, the karyotype of the samples and its possible incidence on viability was studied.

## Results

### Study of *ARG 17* Colony

Analyzing *ARG 17* genetic variability [11], [12], [26]–[28] as a whole, we found associations among cytological, morphological and physiological factors detected over the past 30 years.

*ARG 17* presented morphological mutants. In addition, polymorphisms affecting rostrum pigmentation and thorax vertex basal pattern were observed. Despite efforts to elucidate their genetic control, it was not possible to find a simple explanation for them and it has been suggested that multiple gene effects might have been involved (unpublished).

**Figure 1:**
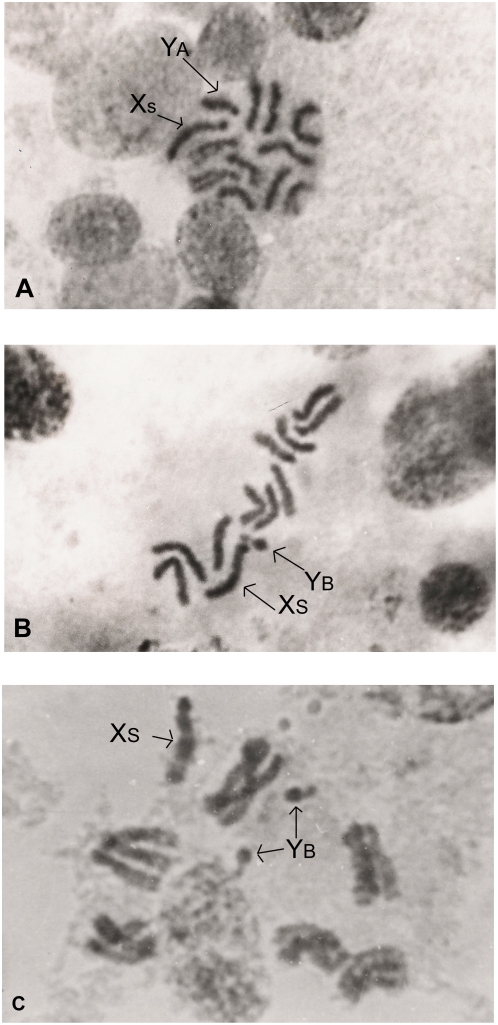
Mitotic metaphase plates of different specimens of the *ARG 17strain*. (a) An X_S_Y_A_ male. (b) An X_S_Y_B_ male. (c) A triploid X_S_Y_B_Y_B_ male, 4000×.

Karyotypical analyses of the colony were carried out throughout the generations. Cytological studies demonstrated the existence of sexual chromosome polymorphisms (Figures 1a, 1b), sexual trisomy, sexual tetrasomy, and triploidy (Figure 1c).

An in-depth analysis of these variations and their genetic consequences can be summarised as follows: the Y chromosome carries the sexual determining factor [26] [28], which is located in the long arm next to the centromere. Y-chromosome variants Y_A_ (Figure 1a) and Y_B_ (Figures 1b, 1c) modified their frequencies within this laboratory strain. At present the *ARG 17* strain only carries the Y _B_ chromosome and is named *ARG 17- Y short*.

So far, no polymorphism of the X-chromosome has been found within *ARG 17*. The acrocentric X-chromosome which is present in *ARG 17* is considered the standard type and is named X_S_ (Figure 1). The X_L_ variant which was found within a familiy originating in the same ecological niche as the *ARG 17* is also acrocentric. Table 1 shows the size ratios between each Y-variant and the Xs-chromosome, as well as between the Y_A_ and the X_L_ and between both X-chromosome variants.

**Table 1:** Size ratios between sexual chromosome variants within *ARG 17*.

Sexual chromosomes	Size ratio
Y_B_/X_s_	0.27±0.011
X_S_/X_L_	0.86±0.027
Y_A_/X_L_	0.55±0.025
Y_A_/X_S_	0.62±0.019

### Experiment I

The *T5038* autosexing strain developed in Buenos Aires germplasm and the *T15879* autosexing strain enriched with *Mendoza 1* germplasm were crossed with Mendoza 1. This was performed to determine whether the shortage of fertile matings previously observed [10] was due to lack of compatibility or other reasons.

Table 2 shows the results of 94 crossings, where “n” in the “matings” column is the actual number of observed matings. The “Type of choice” column indicates the number of matings between the same or different germplasms. Additionally, the type of choice or the mating preference was confirmed by the offspring phenotype analysis. From table 2 we can conclude that when *T5038* males have the possibility to choose among germplasms, they prefer their *T5038* sisters rather than *Mendoza 1* females. When only Mendoza 1 females are available to *T5038* males, no matings are observed. On the other hand, *T15879* derived males from the same translocation, enriched with Mendoza germplasm, mate with both types of females indiscriminately.

**Table 2:** Compatibility screening between Buenos Aires and *Mendoza 1* germplasms (E I).

TYPE OF MATE	Families n	Matings n	Type of Choice
1 male with 2 females			18 with *nig*
♂A×♀A (*nig*) and ♀B(+)	50	20	2 with *Mza.*
1 male with 1 female			
♂A×♀ B (+)	16	0	-
1 male with 2 female			13 with *nig*
♂ C×♀ C *(nig*) and ♀ B(+)	28	26	13 with *Mza*

A = *T/5038*; B = *Mendoza*; C = *T/15879*.

### Experiment II

Different studies were carried out to analyze genetic variability within *Mendoza 2*.

### G_1_ Cytological Screening of *Mendoza 2*

**Figure 2:**
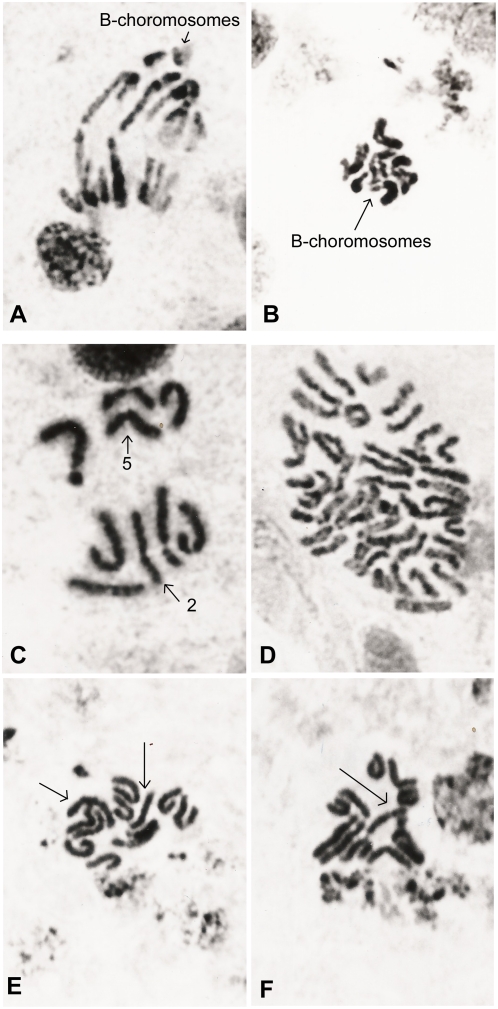
Mitosis in cerebral ganglion cell of different G_1_ specimens of Mendoza 2 colony. (a) Anaphase plate showing B-chromosomes, 5700×. (b) Metaphase plate of female carrying a pair of B-chromosomes, 4000×. (c) Incomplete metaphase plate of female showing chromosomal translocations involving autosomal pairs 2 and 5, 6900×. (d) A polyploid plate from a mosaic individual, 4000×. (e) Metaphase plate of an X_S_X_L_ female. Arrow indicates X_S_ and X_L_ chromosomes, 5700×. (f) Metaphase plate of an X_S_Y_L_ male. Arrow indicates the B-chromosome attached to the Y-chromosome, 5700×.

**Figure 3:**
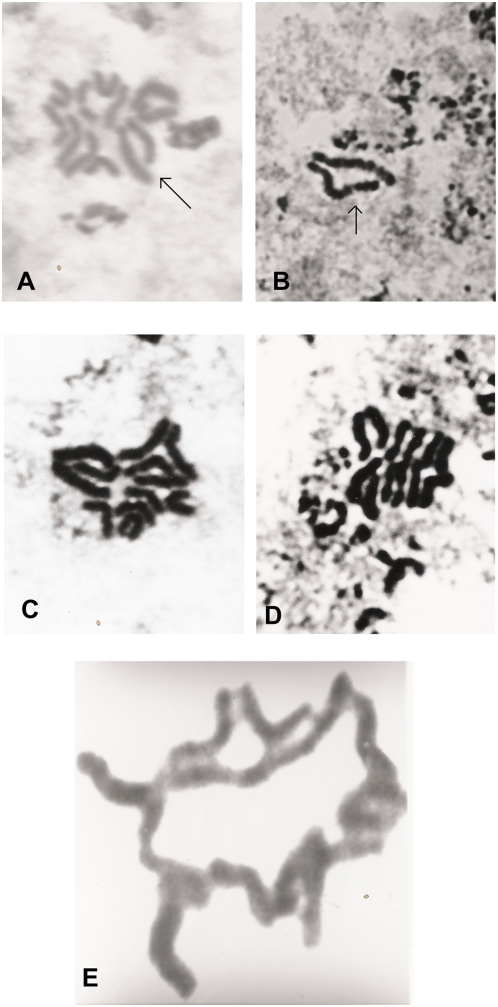
Mitotic metaphase plates of F_1_ specimens from different Mendoza 2 families. (a) A specimen from family 61 carrying a heterozygous inversion affecting pair 3 and reciprocal translocations, 5700×. (b) Heterozygous inversion for pair 3 in a specimen belonging to family 68. Arrow indicates the inverted member of the autosomal pair, 6900×. (c–d) Heterozygote for the reciprocal translocation belonging to families 8 and 27. Arrow indicates the classical cross shape formed by chromosomes involved, 5700×. (e) Ring of chromosomes corresponding to a heterozygote for multiple translocations in family 65, 6900×.

Karyotypic studies of G_1_ demonstrated that *Mendoza 2* is a very polymorphic population, partially different from the *ARG 17* strain. However, *Mendoza 2* shares some features with *ARG 17*. Mutations involving changes in the number and/or shape of the chromosomes (Figures 2, 3) are observed in 76% of the G_1_ individuals (Table 3); we detected accessory or B-chromosomes (Figures 2a, 2b; Table 3), unequal autosomal pairs (Figure 2c, Table 3) and mosaic specimens carrying polyploid metaphases (Figure 2d, Table 4). One or two B-chromosomes are involved in most of these abnormalities, being found in 93% of the F1 individuals (Table 3). They are either free or attached to sexual chromosomes so that they become X_L_ (Figure 2e) or Y_L_ (Figure 2f). We do not know whether all the B-chromosomes have the same origin.

**Table 3:** Cytological analysis of the G1 and F1 offsprings from *Mendoza 2* (E II).

Karyotype	Abnormal (chromosomal mutations)					Normal
	1	2	3	4	Total	Total
% G1 individuals	30	31	15	-	76	24
% F1 individuals	86	-	-	7	93	7

1 = With B chromosomes, 2 = rearrangements, 3 = Mosaic specimens, 4 = Without B chromosomes.

**Table 4:** F1 Fertility and chromosomal mutations from *Mendoza 2* (E II).

Family	F1	
	Rearrangement	Fertility (%)
61	heterozygous inv.	88
68	heterozygous inv.	81
8	reciprocal transloc.	68
27	reciprocal transloc.	69
65	Multiple transloc.	90
94	YL Chromosome	72
63	XL chromosome	45

### F_1_ fertility and chromosomal mutations

**Figure 4:**
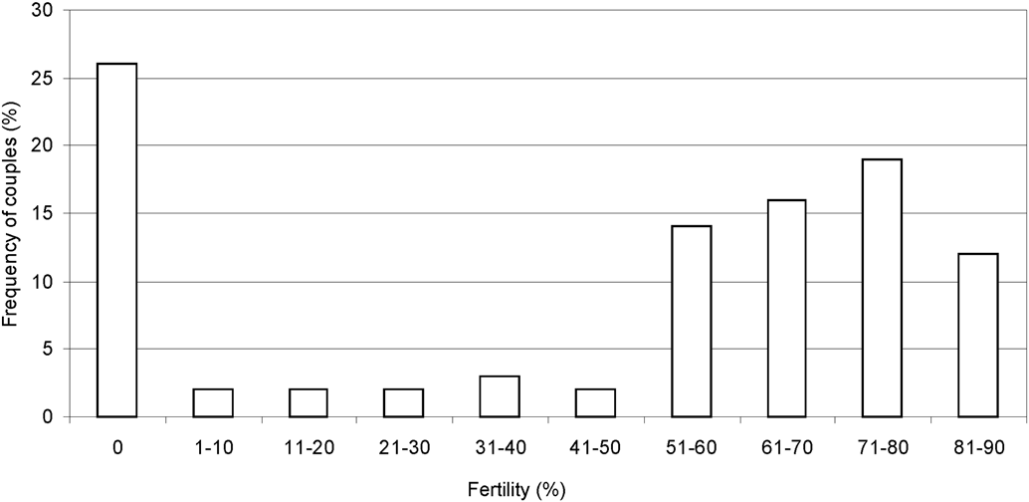
F1 fertility of successful egg-to-pupa development events measured as pupae to egg percentage.

Of the 73 couples originally assembled, 86% oviposited. The mean value of laid eggs per female per day was 39+1.8. The analysis of fertility of the 63 couples that oviposited, measured as pupae percentage out of egg number is represented in Figure 4, and reveals that 75% of the families reached the pupa stage. This means that 47 out of 63 families contributed to the F_2_ generation. Several families showed good F_1_ fertility, although a cytological analysis of their larvae demonstrated the existence of chromosomal rearrangements (Table 4).

For instance, a heterozygous inversion affecting chromosome 3 was present in families 61 and 68 (Figures 3a, 3b), whose fertility rates were 88% and 81% respectively (Table 4); heterozygotes for reciprocal translocations were found in families 8 and 27 (Figures 3c, 3d), but their fertility rates were 68% and 69% respectively (Table 4). Heterozygous multiple translocations were observed in family 65 (Figure 3e), which showed an F_1_ fertility rate of 90% (Table 4). Family 94 carrying the Y_L_ chromosome (Figure 2f) showed a 72% fertility rate, but family 63 carrying the X_L_ chromosome (Figure 2e) shows only a 45% fertility rate (Table 4). The frequencies observed in F_1_ offspring are consistent with those expected when calculated on the basis of the observed G_1_ frequencies of the previous generation (Tables 3, 5).

**Table 5:** Expected F1 karyotypes distribution: G1 parents' frequencies and the resulting F1 frequencies are in accordance to those observed and showed in table 3.

		G1 ♂		F_1_ Frequencies	
		0.24 Normal	0.76 Abnormal	Normal	Abnormal
G1 ♀	**0.24 Normal**	0.06	0.18	0.06	0.94
	**0.76 Abnormal**	0.18	0.58		

### Morphological and physiological mutations: F_2_ segregations of 47 families from *Mendoza 2*

The presence of morphological mutants such as pupa colour, imago colour, or eye colour mutants were detected in 19 families. However, segregations did not adjust to F_2_ values since mutants appeared in smaller numbers than expected (Table 6). The eye colour mutant was observed in four of the families, such as family 61, in which a very low 30% viability was recorded. This mutant was isolated and maintained as a new laboratory stock. Physiological studies of this stock are consistent evidence that its developmental time is longer than that of wild individuals in the same population.

**Table 6:** F2 genetic results of the 47 families from *Mendoza 2* (E II).

28 Families		19 Families		
60%		40%		
**Normal**		**Normal**	**Abnormal**	
	**Pupae variation**	N = 15422	Viable	Inviable
			17%	83%
			N = 211	
	**Imagoe variation**	**Normal**	**Color mutants**	
		N = 9061	N = 253	

### Sex ratios and cytological disorders

**Figure 5:**
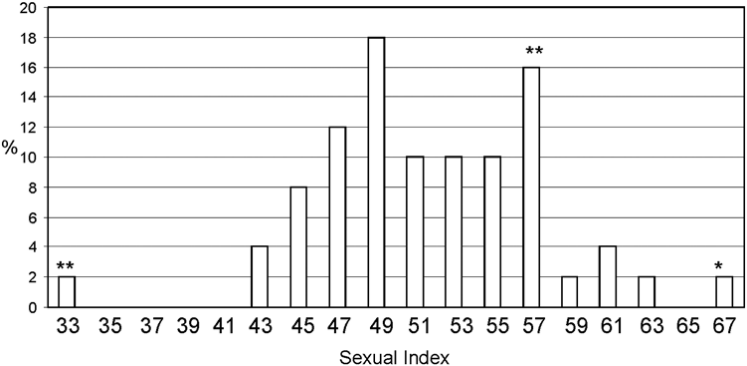
Frequency distribution of sexual index. Extreme values 0.33 and 0.67 correspond to families 94 and 63 respectively. * Families differing from G Test expected values P>5%. ** Families differing from G Test expected values P>1%.

The sexual indexes of each F_2_ offspring is analyzed in Figure 5, where those differing statistically from the expected values are pointed out. Both extremes of the distribution were compared with the corresponding F_1_ cytological analysis (Table 7). An association was observed between distorted sexual ratios and cytological disorders (Table 7). The F_2_ progeny of family 94 showed an imago sex index of 0.33, p>1% (Table 7, Figure 5). The male to female ratio in this family was 61 ♂: 127 ♀. Of the 278 F_2_ pupae, only 188 reached the imago stage. If the missing 50% of males had been among those pupae which did not reach the imago stage, this would have revealed the presence of a Y-linked lethal factor. This is consistent with F_1_ cytological and genetic data, which showed a B-chromosome attached to a Y_A_-chromosome; the Y-chromosome is cytologically observed as an Y_L_-chromosome (Figure 2f). These results suggest that the Y_L_-chromosome would behave as a lethal sexual factor which would be carried by the males and cause them to die during the pupa stage. It should be pointed out that the Y_A_ is not derived from the attachment of a B-chromosome to Y_B_.

**Table 7:** Association between F2 sexual index deviations and F1 altered sexual chromosomes from *Mendoza 2* (E II).

Family	F1 viability	F2			Sexual	F1
	egg-pupae	Pupae	Adults		index	Cytological
	%	N	N♂	N♀	♂ F_2_/total	analysis
94	72	278	61	127	0,333**	Y+B
63	45	158	65	32*	0,670 *	X+B

G test * P>5% G test ** P>1%.

On the other end of the distribution, the F_2_ progeny of family 63 shows an imago sexual index of 0.67, p>5% (Table 7, Figure 5). The male-to-female ratio in this family was 65 ♂: 32 ♀. Of 158 F_2_ pupae, only 97 reached the imago stage, which would point to the presence of a sex-linked lethal factor. The cytological analysis of the F_1_ offspring showed zygotes carrying a B-chromosome attached to the X-chromosome. This sexual chromosome is cytologically observed as an X_L_ (Figures 2e, 5). Since we did not find X_L_X_L_ zygotes, this could account for the female lethality.

## Discussion

Results indicate that the Buenos Aires population is partially different from the Mendoza population. Genetic analysis showed that both laboratory colonies are a reservoir of mutational and cytological polymorphisms which are responsible for partial reproductive incompatibility.

### Karyotypic variability within colonies

Karyotypic polymorphisms within the *ARG 17* colony are maintained and transmitted from parents to offspring. Changes in Y-chromosome variant frequencies were recorded through the years. Within rearing facilities, founder populations of fruit flies are under human management and controlled conditions. Thus, their life cycle is closer to that of microorganisms than to the standard cycle of the species in the natural population. This situation makes it possible to detect the reaction of colonies to new environmental conditions. Most of the phenotypes within a population need genetic plasticity in order to overcome environmental changes during development. Hidden genetic plasticity within founder populations can be detected if recombinations of their variants take place.

Both *Mendoza 1* [10] and *Mendoza 2* colonies also revealed the presence of chromosomal polymorphisms. A high proportion of F_1_ offspring derived from *Mendoza 2* couples showed rearrangements. In our laboratory, the analysis of *C. capitata* polytene chromosomes demonstrated spontaneous inversions and translocations in the Mendoza population [15]. Heterozygous genotypes maintain developmental homeostasis, which allows them to adjust to environmental changes. Position effects along with gene mutations represent a source of genetic variation. Frequently, chromosomal rearrangements become associated with position effects as a consequence of a change in the order of genes [29].

### Cytological disorders, viability and distorted sexual ratios

Both our cytological study and the viability study reflect a representative sampling of the families and the genetic transmission of chromosomal mutations (Tables 3, 5). Some sexual chromosome variants, such as the X_L_ and the Y_L_, behave as a sex-linked lethal factor and are responsible for sexual ratio distortions. Female lethality within family 63 could be explained by the presence of the X_L_-chromosome. This lethal mechanism had been postulated [11] previously during a study of the Buenos Aires germplasm. A similar phenomenon has been detected in the Mendoza population, in which this chromosome modifies the insect viability. Similarly, the Y_L_-chromosome could account for male lethality within family 94 (Table 7). The theoretical analysis [30] of sex-linked meiotic drive found four types of sex chromosomes segregating in some populations, and cycling of frequencies was proposed as a result. An X-chromosome polymorphism due to a driving X-chromosome (X (D)) which causes linkage imbalance has been reported in *Drosophila recens* [31].The eye colour mutant within family 61 provided evidence of being a morphological marker for longer developmental time. Slow development was isolated from family 61, which is a physiological mutant caused by a conditioning lethal allele of the *sw* gene called *sw^x^* [32], [33]. The segregation distortion situation was attributed to the *sw* gene and detected in the eye colour mutant study in F_2_ segregations. As the *sw* gene is lethal in some conditions but not in others – *sw* mutants specially need managing conditions to develop – we suggest that they are the cause of the lack of F_2_ adjustment in some families such as 61. Generally, populations retain hidden recessive genetic variation through a great deal of alleles – masked by a single normal allele – in the heterozygous state. Crossings within these populations help to reveal recessive alleles in the homozygous state and, consequently, identify variability. In many cases, those alleles show less fitness and even depression in the homozygous state [34], [35]. However, they are sources of re-adaptation to environmental changes. In some other F_2_ distorted segregations, it was not possible to describe the genetic control mechanism. Segregation distorters are difficult to observe unless detectable genetic markers are present and unless a driving element occurs polymorphically [31], [36]. A mobile DNA insertion in *D. simulans* was suggested as a possible source of adaptive change [37] and new hypotheses were proposed regarding the mechanisms controlling polymorphisms [38]. The medfly genome contains a rich assortment of transposable elements which display different levels of diversity, abundance and distribution [22]. The presence of actively transposing elements in its genome is revealed by hybrid dysgenesis phenomena, which include a range of abnormalities, insertion site polymorphisms and other genetic instabilities [22]. These phenomena are the result of the movement of transposable elements after hybridization between individuals that possess different complements of transposable elements. Furthermore inter- and intra-strain polymorphism in insertion sites suggests that active copies of some elements such as the *cchobo* element may be transposing in the medfly genome [21].

Structural heterozygosity becomes more or less enforced when lethal genes are included in the chromosome complement [39]. Markers within the inversions show patterns of gametic imbalance, implying little or no recombination between inverted regions [40]. Family 61 is a good representative of this phenomenon since it also carries an inversion in the heterozygous state, showing 88% fertility (Table 4) but 30% viability. The pattern of imbalance also suggests that alternative rearrangements may contain beneficial co-adapted suites of genes [40], such as family 65 displaying rings of variable numbers of chromosomes (Figure 3e), its fertility rate being 90% and its viability rate 56%. Partial heterokaryotype sterility seems plausible because there is plenty of evidence that heterozygotes for inversions, translocations and tandem fusions produce gametes with deficiencies and duplications [41].

### Reproductive barriers within colonies

Cytological study confirmed the presence of chromosomal polymorphisms within the *Mendoza II* colony affecting the insect viability: 14% of the couples did not oviposite, thus suggesting the existence of pre-zygotic isolation mechanisms. In the case of that 25% of families whose eggs could not reach the pupa stage (Figure 4), it must be considered whether those eggs were fertilized or not. If fertilization took place, then post-zygotic mechanisms such as segregational sterility would be responsible for isolation barriers, but if this were not the case, we would still have to consider pre-zygotic barriers. In the case of those families carrying cytological abnormalities, it is likely that many of the pupae could not reach the imago stage (Table 6). Chromosomal rearrangements can promote reproductive isolation by reducing recombination along a large section of the genome [41]. Pre-zygotic mechanisms should be favoured by natural selection, but post-zygotic mechanisms should be the product of genetic divergence. The biological function of chromosomal polymorphisms in translocations and inversions is probably the same: establishment of linkage imbalances [42] and supergenes of adaptive value [43].

### Genetic incompatibility between Buenos Aires and Mendoza germplasms

Experiment I demonstrated the existence of incompatibility between Buenos Aires and Mendoza germplasms, showing isolation barriers between them. We provided consistent evidence that polymorphisms are responsible for lack of compatibility. Genetic isolation is caused mostly by translocations and inversions. We believe that these barriers are due to sexual or ethological pre-zygotic mechanisms. There are unforeseeable factors which man cannot manage: bottle-necks that reduce genetic variability and phenotypes that cannot adapt to colonization. This phenomenon – a form of genetic drift – is only possible when genetic variability exists [29].

A program to eradicate the screw-worm (*Cochlyomia hominivorax*) in the United States, based on the release of sterile blowflies, failed because a state of reproductive incompatibility developed between wild-type and laboratory-reared individuals. A chromosomal polymorphism affecting the genital morphology of wild type females was associated with isolation barriers [24]. Similarly, the re-invasion of California by the medfly (*Ceratitis capitata*) and the failure to control it [44] was due in part – as with the screw-worm – to changes in the composition of that particular population.

A test of the sterile insect technique program against the medfly in coffee plantations of Kauai, Hawaii, failed because native females altered their mating preferences, rejecting most laboratory-reared males during courtship [45]. In outdoor field cage experiments, these authors demonstrated that females from other non-treated Hawaiian islands did not change their mating preferences over the same period and accepted laboratory males 5–10 times more often than resistant Kauai females did. The sexual isolation between mass-reared strains and wild materials of the Medfly was measured [46] in order to know if this parameter can be used to decide which strain is more suitable for field release. Later on, significantly more mating was found in tests involving wild flies of a particular Australian population [47].

Visible mutations are only part of the entire genetic variability, which includes polymorphisms. Because heterozygous individuals are partially sterile, these chromosomal changes act as genetic barriers and are probably the cause of incompatibilities between populations. As these polymorphisms can limit intercrossing between different populations at any time, they cannot be ignored. Incompatibility between the translocated laboratory strain *T5038* and *Mendoza I* laboratory population can be solved by producing the translocation mechanism and the marker mutant in the germplasm of the population to be controlled. This would be the most immediately effective measure to avoid isolation barriers between populations in control programs based on genetic strategies.

The auto-sexing mechanism represents an improvement on the SIT technique since males and females can be recognized at immature stages. Then, only male pupae will be sterilized for control purposes, since females are eliminated during rearing. This is relevant because adult females, although sterile, maintain their oviposition habits. The auto-sexing mechanism avoids the unnecessary increase in a) the number of females in the population and b) damage to fruits. Another improvement on SIT-based control programs was the construction of transgenic strains of the medfly harbouring a tetracycline-repressible transactivator (tTA) that causes lethality in the heterozygous progeny [3]. This dominant lethal genetic system avoids the problems of radiation-sterilization, but it must indeed be developed in the population to be treated so as to avoid putative genetic incompatibilities among germplasms.

### Conclusions

Present data provide consistent evidence that, in order to avoid pre-zygotic isolation barriers between target and laboratory populations, the genetic control mechanism should be produced directly in the treated population's germplasm. This is an additional condition for the success of the control programs based on genetic strategies such as the Sterile Insect Technique for controlling *Ceratitis capitata* populations. Additionally, we discovered natural auto-control mechanisms, such as the sex-linked lethals causing distorted sexual ratios.

The periodic study of colonies reveals precious information on naturally occurring control mechanisms such as those detected within the colonies, which could be used by geneticists in order to modify pest population dynamics.

## Materials and Methods

Table 8 summarises the materials used in this work along with the methods and experiments performed to study Mendoza colonies.

**Table 8:** Materials and Methods.

EXPERIMENTS	MATERIALS		CYTOLOGY	MORPHOLOGY			PHYSIOLOGY	CROSSINGS
				Pupa	Imago			
					Body	Eyes		
E I	Mendoza I			X	X			X
	strain T 5038			X	X			X
	strain T 15879			X	X			X
E II		G1	X	X	X	X		
	Mendoza II	F1	X				X	
		F2	X	X	X	X	X	

### Materials

Materials were maintained following the technique described by Terán [48], which is our routine rearing technique.

#### *ARG 17* Colony

It was originated in 1965 at the Institute of Plant Pathology (Eng. Turica) with samples from San Pedro (Long. 59.41; Lat. 33.41) and samples from the area around Castelar (Long. 58.39; Lat. 34.40), both of which localities are in the Province of Buenos Aires. This material was used in the SIT control programmes in the original area. It received recurrent introductions of wild material from the same areas (Eng. Turica, personal communication). A sample of 2,428 pupae (mean weight = 9.614 mg/pupae) was carried to the Insect Laboratory (I.G.E.A.F.) in 1973. A bottle-neck was observed during the following generation: of over 55,000 laid eggs, only 9,830 imagines, or 20%, were recovered. Of these, over 30% died during the first three days. The remaining imagines gave rise to *ARG 17*, which has been maintained as a closed population, and to date (35 years later), no inbreeding problems have been observed. It is at present the reference base material of the laboratory. Some data about this strain have already been reported and, in this paper, they have been summarised for easier understanding.

#### Mendoza Founder Populations

Mendoza founder populations represent two colonies founded with specimens taken from different host-fruits and localities of Mendoza (Long. 68, Lat. 37) which received recurrent introductions throughout successive generations. The colony used in our experiments during 1986 will be referred to as *Mendoza 1*, and the other one used in 1994 as *Mendoza 2*. These colonies were also employed in pioneering studies for control programmes (SIT) in that province.

*Mendoza 1* was a sample of approximately 6,000 pupae taken from the Mendoza laboratory population. *Mendoza 2* was a sample of approximately 28,000 pupae received from the Mendoza laboratory population (G_0_).We assembled 73 F_1_ families from the *Mendoza 2* colony. A “family,” as termed by Lerner [49], was founded from single-pair matings by randomly taking males and females from G_1_.

#### Auto-sexing Strains

The *T5038 Y+/X nig* strain [50], [32] resulted from a translocation from autosome 2 to the Y_A_ sexual chromosome. Autosome 2 carries the cuticular marker *nig (niger)*, a recesive black pupa and imago mutation [8]. This Y-autosomic translocation was produced in *ARG 17* germplasm 100 generations ago. All the females are homozygous for the marker nig (black pupal and imago phenotype = *niger* females) and all the males are wild-type (wild pupal and imago phenotype), because they are heterozygous for the *niger* gene. In the present work, this auto-sexing strain was used to measure compatibility with Mendoza germplasm. The advantage of having an auto-sexing strain is that this material makes it possible to recognize and separate males from females in immature stages such as the pupal stage and avoid releasing sterile females.

#### Auto-sexing Strain on Mendoza Germplam

The *T15879 Y+/X nig* Strain (with Mendoza germplasm) was used to reconstitute the auto-sexing mechanism of the *T5038* strain made up in the Mendoza germplasm.

#### Compatibility crossings

We assembled 94 families in the following way:

50 families: 1 male *T5038*×1 female *T5038*×1 female *Mendoza I*
16 families: 1 male*T5038*×1 female *Mendoza I*
28 families: 1 male *T15879*×1 female *T15879*×1 female *Mendoza I*

### Study of the *ARG 17* Colony

A morphological and cytological analysis of the reference laboratory strain *ARG 17* was performed. The chromosomal constitution of flies was periodically determined from 1973 up to now. Morphological studies were performed on pupae and imagines, analyzing colour segregation, rostrum pigmentation and thorax vertex basal pattern in both sexes, number of spatulated hairs in males, and number of orbital rostrum hairs in females.

### Experiment I

A test of compatibility of the translocated *T5038* and *T15879* strains with *Mendoza 1* germplasm was performed in order to use an autosexing strain to control Mendoza wild population. The number of matings was studied in order to determine the male preference. The offspring colour segregation in the pupa and adult stages was analyzed in order to determine its maternal origin. Using this method, the offspring from crossings with female *T5038* should be black and the offspring from crossings with Mendoza females should be wild type. For this purpose, 94 families were assembled in the following way: 50 families with one male *T5038 (+)* and two females, *T5038 (nig)* and *Mendoza 1 (+)*; 16 families with one male *T5038 (+)* and one female *Mendoza 1 (+)*; 28 families with one male *T15879 (+)* and two females, *T15879 (nig)* and *Mendoza 1 (+)*.

### Experiment II

The analysis of genetic variability within the *Mendoza 2* colony was conducted on samples of the G_1_ and on the F_1_ and F_2_ progenies of 73 assembled families, studying karyotypes and physiological and morphological characters.

#### Cytological Study

We conducted the karyotypical study on a G_1_ larvae sample (N = 13) of *Mendoza 2* and on 36 derived laboratory strains (N = 81). We obtained cytological data from 1 to 4 individuals out of 5 chromosome spreads belonging to 5 specimens per strain.

#### Cytological Techniques

The chromosomal constitution of the flies was determined through the cytological analysis of mitotic metaphases in the cerebral ganglion cells from third instar larvae. Ganglion cells were stained with 2% lacto-propionic orcein for 5 hours at 25°C, as described by [9]. Data were obtained from the analysis of at least 10 metaphase plates per chromosome spread.

The sexual chromosome variant size ratios were calculated measuring the length of a pair: the shorter chromosome variant against the other one. The relative chromosome length was the mean value obtained from repeated measurements of at least 10 different metaphase plates within each chromosome spread of a larvae sample.

#### Physiogenetic Study

The first egg-laying opportunity and fertility measured through F_1_ egg-hatching were tested. Those couples which did not lay eggs the first time were given a consecutive second opportunity to be tested.

#### Viability and Sex Ratio

The F_2_ from *Mendoza 2* was analyzed through the study of their pupal viability, sex ratio and spontaneous segregation of mutants.

For the statistical analysis of the sexual index, the G-test with a null hypothesis for a sex ratio of 50∶50 was used [51], [52]. The sexual index of each family was calculated as the number of males out of the total number of individuals.

Cytological and physiological data were compared.

#### Morphological study

Pupae and imago from G_1_ and F_2_ segregations were analyzed in terms of pupa colour, imago colour, or eye colour mutants.This section should provide enough detail to allow full replication of the study by suitably skilled investigators. Protocols for new methods should be included, but well-established protocols may simply be referenced. We encourage authors to submit, as separate supporting information files, detailed protocols for newer or less well-established methods. These are published online only, but are linked to the article and are fully searchable.
